# *De novo* Assembly and Genome-Wide SNP Discovery in Rohu Carp, *Labeo rohita*

**DOI:** 10.3389/fgene.2020.00386

**Published:** 2020-04-21

**Authors:** Paramananda Das, Lakshman Sahoo, Sofia P. Das, Amrita Bit, Chaitanya G. Joshi, Basdeo Kushwaha, Dinesh Kumar, Tejas M. Shah, Ankit T. Hinsu, Namrata Patel, Siddhi Patnaik, Suyash Agarwal, Manmohan Pandey, Shreya Srivastava, Prem Kumar Meher, Pallipuram Jayasankar, Prakash G. Koringa, Naresh S. Nagpure, Ravindra Kumar, Mahender Singh, Mir Asif Iquebal, Sarika Jaiswal, Neeraj Kumar, Mustafa Raza, Kanta Das Mahapatra, Joykrushna Jena

**Affiliations:** ^1^Fish Genetics and Biotechnology Division, ICAR-Central Institute of Freshwater Aquaculture, Bhubaneswar, India; ^2^Department of Animal Biotechnology, Anand Agricultural University, Anand, India; ^3^Molecular Biology and Biotechnology Division, ICAR-National Bureau of Fish Genetic Resources, Lucknow, India; ^4^Center for Agricultural Bioinformatics, ICAR-Indian Agricultural Statistics Research Institute, New Delhi, India; ^5^Division of Fisheries, Krishi Anusandhan Bhawan - II, New Delhi, India

**Keywords:** rohu carp, draft genome, *de novo* assembly, orthologous gene family, synteny, phylogenetics, otophysan

## Introduction

Carps constitute a very large group of freshwater fish belonging to the family Cyprinidae, and are predominant in aquaculture system accounting for ~71–75% of freshwater fish production (FAO, 2018). The largest producer of carp is China (78.7%), followed by India (15.7%); the remaining is produced by Bangladesh, Myanmar, Vietnam, Indonesia and Pakistan collectively, contributing more than 30% of global aquaculture production in terms of tons (FAO, 2017a). Among the three Indian major carp species (*Labeo rohita, Catla catla* and *Cirrhinus mrigala*), rohu carp (*L. rohita*) is the most popular due to its growth potential coupled with high consumer preference. The natural habitat of this species is the Indo-Gangetic riverine system, encompassing northern, eastern and central India, as well as the rivers of Pakistan, Bangladesh and Myanmar. The species has also been introduced in many other countries, including Sri Lanka, the former USSR, Japan, China, the Philippines, Malaysia, Nepal and some African countries. The traditional culture of rohu in the small ponds of the eastern Indian states dates back hundreds of years. *L. rohita* currently accounts for ~2.5% of total freshwater aquaculture production worldwide (FAO, 2017b). The Central Institute of Freshwater Aquaculture, India, has established a selective breeding programme for rohu carp with the aim of increasing the growth rate of this fish from 700 to 1,000 g in a year (Chondar, 1999) to more than 1,500 g a year. A genetically improved strain of rohu, called “Jayanti,” was developed. A 17% higher average growth rate per generation was achieved after four generations of selection (Das Mahapatra et al., 2006). Our previous studies have also reported DNA markers such as SSRs (Das et al., 2005; Patel et al., 2009; Sahu et al., 2012), SSR and SNP -based linkage maps (Robinson et al., 2014; Sahoo et al., 2015) and transcriptome resources (Robinson et al., 2012; Sahu et al., 2013) in this species. However, whole genome sequence of rohu carp is still lacking. In this study, we present the first draft genome of rohu to complement the on-going selective breeding program by generating genomic resources. Besides, the genome information can be useful for functional and comparative genomics, gene mapping, genome wide association, and genomic selection studies. With the advancement of sequencing technologies, there has been a rapid increase in the number of genome assemblies for terrestrial species compared to aquatic species (including fish) in the last decade, with a very small (Kelley et al., 2016) percentage of fish genomes given the most numerous taxonomic group and huge diversity exhibited by teleosts (Ravi and Venkatesh, 2018). The representation of carps in the genome database is further very limited.

Rohu carp is a member of Otophysi, a major clade of modern freshwater fishes. The superorder Otophysi is currently classified into four orders: Cypriniformes (carps and minnows; 4,262 species), Characiformes (tetras and piranas; ~2,100 species), Gymnotiformes (knifefishes and electric eel; 225 species) and Siluriformes (catfishes; ~3,700 species) (Eschmeyer and Fong, 2015; Nelson et al., 2016). Despite significant advances recently in delineating lineages within major taxonomic groups at the molecular level, an increasing number of whole-genome sequences of fish may be needed to address the evolution of otophysan lineages and the phylogenetics at the genome scale. Within this context, the genome sequence of rohu carp would provide an essential resource for evolutionary and biological studies in addition to carp genetic improvement.

Adopting the whole-genome shotgun protocol and a multi-platform sequencing approach, we for the first time generated a good quality genome assembly of rohu carp. By resequencing ten wild populations, we have also identified approximately five million SNPs. Additionally, we also performed phylogenetic analysis of rohu and thirteen other otophysan species to determine the phylogenetic position of rohu within otophysan lineages.

### Value of the Data

Here we report for the first time the draft genome of Indian major carp, rohu widely cultured in Indian subcontinent. The scaffold N50 was found to be 1.95 Mb and there were 26,400 protein coding genes and 40.63% repeats.

Resequencing of 10 riverine rohu populations identified ~5 million SNPs which will provide a valuable resource for undertaking genome wide association studies, genomic selection, population genomics and fine-mapping of QTLs in this species.

Phylogenetic analysis taking protein sequences of 335 single copy genes of 14 Otophysans revealed that rohu carp (Labeoninae) was at a position equidistant to the other species in the Otophysi clade, forming a sister group.

All the six families and four subfamilies under the four otophysan lineages were monophyletic.

## Materials and Methods

### Genome Sequencing

A single male rohu (~1 kg), belonging to seventh generation of ongoing selective breeding programme of ICAR-CIFA, was chosen for sequencing. Tissue samples were collected in September 2013. All handling of fish was carried out following the guidelines for control and supervision of experiments on animals by the Government of India and approved by Institutional Animal Ethics Committee (AEC) of ICAR-CIFA. The fish was anesthetized followed by harvesting of the testes, liver and muscle tissues, and isolation of high- molecular weight genomic DNA using standard phenol-chloroform extraction method (Sambrook et al., 1989). A multi-platform sequencing strategy was adopted to generate approximately 130-fold coverage sequence data for the estimated genome size of 1.5 Gb. Approximately 1,000 ng of genomic DNA per library was sheared using a Covaris S2 sonicator (Covaris, Woburn, Massachusetts, USA) to generate fragments ranging in size from 200 bp to 20 kb. A total of 18 libraries (single-end, paired-end and mate-paired) including one large insert library (Supplementary Table 1) were prepared for Roche 454 (GS FLX), Illumina (Miseq and Nextseq 500), Ion Torrent (PGM), and PacBio (Sequel) sequencing using respective protocols. Briefly, Roche libraries were prepared and sequenced using picotitre plates with Titanium or long-read chemistry (Roche Diagnostic, USA). Illumina Miseq libraries were prepared using the Nextera XT library prep kit and Illumina Nextseq 500 libraries were constructed following the TruSeq PCR-free HT library Prep Kit. In addition, one shotgun library for Ion-Torrent PGM and one large insert (15–20 kb) library for the PacBio (Sequel) platform were prepared following the manufacturer's instructions.

### *De novo* Genome Assembly and Validation

The raw sequence data were checked for quality using FastQC and the NGSQC (NGSQC Patel and Jain, 2012). Low quality (Q <20) and short (<50 bp) reads were filtered out to obtain a set of usable reads. The assembly was obtained using the MaSuRCA assembler (Zimin et al., 2013). First, all data except for PacBio data were assembled using MaSuRCA, followed by scaffolding in SSPACE v3.0 (Boetzer et al., 2010). Gap closing was performed using GapCloser v1.12b, a part of SOAPdenovo2 (Luo et al., 2012). Second, PacBio reads were error corrected by Illumina paired-end data using pacBioToCA module implemented in Celera Assembler (Myers et al., 2000), followed by assembly in the CANU assembler v1.7 (Koren et al., 2017). Finally, the gap-closed scaffolds from both analyses were merged using Quickmerge (Chakraborty et al., 2016) (Supplementary Figure 1). Scaffolds more than 2 kb in size were retained to construct the final set. Further, the genome size of rohu was estimated by using the program Jellyfish as implemented in MaSuRCA. The completeness of the genome assembly was assessed using BUSCO version 3.0 (Simão et al., 2015) and Actinopterygii odb9 dataset having a set of 2,586 highly conserved core eukaryotic genes. In order to check the possible redundant sequences in the assembly, the k-mer distribution graph for the complete assembly was generated using jellyfish followed by a 21-mer profile using the Illumina PE reads. Further, the Illumina PE reads were mapped to assembly sequences for analyzing depth distribution for every base in the genome. The accuracy of the assembly was evaluated by anchoring the scaffolds onto published SNP and microsatellite marker maps for rohu (Robinson et al., 2014; Sahoo et al., 2015). For this, SNPs and microsatellite markers of rohu were used as queries against rohu scaffolds by Blastn module as implemented in the program CLC Bio workbench version 7.0.4, with the following parameters: e-value 1e-10, word size 10, match 2, mismatch −3 and % identity 90%.

### Genome Organization

SSRs were screened from the genome using MISA software (Thiel et al., 2003). Repeat identification in the assembled genome of rohu was carried out by homology-based and *de novo* methods. We performed homology-based identification using RepeatMasker version 4.0.6 against *D. rerio* RepBase version 20.07 as the repeat library. The *de novo* repeat library was constructed using RepeatModeler version 1.0.10 which essentially uses two repeat-finding programs, RECON (Bao and Eddy, 2002) and RepeatScout (Price et al., 2005), along with Tandem Repeat Finder (Benson, 1999). The consensus sequences yielded were used as repeat library to mask repeats using RepeatMasker with default parameters. Transfer RNAs were screened across the genome using tRNA scan-SE (Lowe and Eddy, 1997).

### Gene Prediction and Functional Annotation

We carried out combined annotation methods using *de novo*, homology-based as well as transcriptome-based approaches to annotate the rohu genome. The program AUGUSTUS version 3.2.3 (Stanke and Waack, 2003) was used for *de novo* prediction of protein coding genes from the repeat masked rohu genome assembly. RNAseq data derived from various tissues of rohu (generated in this study and available online) were used to support the prediction of proteins by mapping *de novo* assembled transcripts to the genome assembly. In homology-based predictions, putative genes were predicted using trained zebrafish model. We filtered out sequences <100 amino acids from the total predicted protein-coding genes, followed by a Blastp search against the NCBI non-redundant database with default parameters. From the resultant hits, partial and fragmented predictions were checked and removed by performing Blastp against well characterized protein sequences of zebra fish for the final set (Supplementary Figure 2). Functional assignment of the final set of predicted protein sequences was carried out by BLAST2GO v5.0 (Conesa et al., 2005).

### Comparative Genome Analysis

To describe orthologous relationships for the rohu annotated genes, we compared them employing OrthoVenn (Wang et al., 2015) with three other diploid cyprinid species, *Anabarilius grahami, Ctenopharyngdon idellus*, and *Danio rerio*. Orthologous genes shared among these species were depicted through a Venn diagram. Moreover, to reveal the synteny conservation between rohu and zebrafish, the rohu genome sequence was compared with 25 chromosomes of the well-characterized zebrafish genome using Symap v3.4 (Soderlund et al., 2011).

### Whole-Genome Resequencing and SNP Discovery

Resequencing of 10 wild populations of rohu, covering different geographical regions of India, was performed using the Illumina NextSeq 500 platform. The 10 different populations originated from the five Himalayan riverine systems encompassing northern, eastern and central India, and five peninsular riverine systems covering southern India. We sampled 3 individuals from each population and pooled their DNA for paired-end Illumina sequencing. The VDAP-GUI pipeline (Menon et al., 2016) was used for genome wide SNP discovery. Commonly used linux command (head—number of reads “filename.fastq” > “filename.fastq”) was used to extract the number of reads equivalent to the sample having lowest number of reads and then the data were pooled together to make one dataset for mapping against draft genome. The data and reference sequence were then imported into the pipeline, which included quality control by FastQC version 0.11.2 (www.bioinformatics.babraham.ac.uk/projects/fastqc/), quality filtering by PRINSEQ version 0.20.4 (prinseq.sourceforge.net/), and trimming with minimum quality scores of Q20 and sequence lengths of 30 bp. For reference mapping, the BWA-mem version 0.7.5a algorithm was used with the following parameters: match score 1, penalty for mismatches 4 and gap open penalty 6. The SNP/INDEL detection methods used in VDAP-GUI were SAMtools version 0.1.19, VarScan version 2.3.7, and FreeBayes version 0.9.10-3. A custom approach, namely, MultiCom that performs variant discovery using all the above three algorithms was also used. Final SNPs were identified by at least two algorithms. Duplicate removal was performed using the Picard tool (version 1.7.0) (https://broadinstitute.github.io/picard/).

### Phylogenetic Analysis

Phylogenetic relationships were deduced by the maximum likelihood method, based on the protein sequences of 335 single-copy genes (Supplementary Data) commonly shared by fourteen otophysan species representing all four orders, Cypriniformes (8), Characiformes (2), Gymnotiformes (1), and Siluriformes (3). We downloaded the protein sequences of *A. grahami, C. auratus, C. carpio, D. rerio, Sinocyclocheilus anshuiensis, S. graham*, and *Sinocyclocheilus rhinocerous* (Cypriniformes, including rohu), *Astyanax maxicanus, Pygocentrus nattereri* (Characiformes), *Electrophorus electricus* (Gymnotiformes), and *Ictalurus punctatus, Pangasianodon hypophthalmus* and *Tachysurus fulvidraco* (Siluriformes) from the database. These protein data sets were clustered to identify orthologous gene families with ProteinOrtho (Lechner et al., 2011). Three hundred thirty five single-copy genes, common to all the above species, were selected from the clusters for alignment using the software MUSCLE (Edgar, 2004) with default parameters. The individual sequence alignments were concatenated, and gaps were removed before constructing the maximum likelihood phylogenetic tree using RAxML (Stamatakis, 2014) employing PROTGAMMAJTT model with 20,000 iterations toward convergence of the maximum likelihood model and 1,000 bootstrap replicates. Tree viewer was used for viewing the phylogenetic tree.

## Results and Discussion

### Genome Assembly and Validation

The haploid rohu genome containing 25 chromosomes (Zhang and Reddy, 1991) was observed to have an estimated genome size of 1.5 Gb, which is similar to the lengths of male and female genome maps reported in an SNP-based linkage map of rohu (Robinson et al., 2014). The assembly resulted in 259,627 contigs and 13,623 scaffolds, with contig N50 and scaffold N50 values of 30.6 kb and 1.95 Mb, respectively (Table 1). The assembled genome size of *L. rohita* is 1.48 Gb, accounting for >98% of the estimated rohu genome size of 1.5 Gb. In total, 393 scaffolds of 13,623 were found to be more than 1 Mb in size. The draft assembly presented here is of good quality and comparable to other published teleost genomes of similar size (Supplementary Table 2). The rohu draft genome provides a proxy for genome completeness based on 2,586 BUSCOs, which includes 2,472 [95.6%] “complete” BUSCO genes, 1,667 [64.5 %] single-copy, 805[ 31.1%] duplicated, 19 [0.7%] fragmented and 95 [3.7%] missing BUSCOs. The k-mer distribution and depth coverage profiles generated indicated very less or no redundant sequences in the assembly (Supplementary Figures 3–17).

**Table 1:** Assembly statistics of rohu draft genome.

**Parameters**	**Contigs (bp)**	**Merged all** **scaffolds (bp)**	**After gap closing (bp)** **(Length > 2,000 bp)**
Total number	259,627	147,061	13,623
No. of bases	1,236,201,637	1,563,356,456	1,484,730,970
Max. size	12,383,302	15,225,768	15,225,769
N50 value	30,672	2,123,649	1,959,535

We assessed the accuracy of the assembly by anchoring sequences onto the SNP and SSR-based genetic maps of rohu (Robinson et al., 2014; Sahoo et al., 2015). All SNP markers (3,193) with the sequence information matched at unique positions in 667 scaffolds, covering approximately 80% of the genome (Supplementary Table 3). The 667 scaffolds, totaling 1.18 Gb were spread across 1,416 cM of the genome, which was in agreement with the linkage groups of rohu. Similarly, 146 SSR loci covering 25 linkage groups of rohu were also matched (Supplementary Table 4).

### Genome Organization

RepeatModeler was employed for *de novo* repeat modeling, and repeats were found to constitute 40.63% of the rohu genome. Of these, 34.11, 3.9, and 2.32% were interspersed repetitive DNA, satellite DNA and simple repeats, respectively (Supplementary Table 5). The GC percentage (36%) found in this study is similar to that of the genomes of other cyprinids (Supplementary Table 6). The overall percentage of repeat elements observed was similar to the repeat contents of the cavefish *Sinocyclocheilus grahami* (Yang et al., 2016) and grass carp *Ctenopharyngodon idellus* (Wang et al., 2015), higher than common carp *Cyprinus carpio* (Xu P. et al., 2014) and blunt snout bream *Megalobrama amblycephala* (Liu et al., 2017) but lower than zebrafish *Danio* rerio (Howe et al., 2013) (Supplementary Table 6). The most abundant repeat elements in the rohu genome were found to be DNA transposons, accounting for 33.58% of the classified elements, followed by retrotransposons (6.1%), LINEs (3.5%), and SINEs (0.8%), as observed in other carp genomes. Searching for genome-wide simple sequence repeat markers of the assembled rohu genome resulted in 557,193 SSRs, with dinucleotide repeats being the most abundant (Supplementary Table 7).

### Gene Prediction and Functional Annotation

The rohu genome is predicted to contain 26,400 protein-coding genes; 2,516 tRNAs (2,292 tRNAs for standard amino acids, 3 selenocysteine tRNAs, 39 undetermined isotypes, and 182 predicted pseudogenes) were predicted using tRNAScan-SE. More than 85% of the predicted genes were supported by rohu transcriptome data as well as protein database. The number of genes predicted for rohu is similar to that for other diploid cyprinids, such as zebrafish, blunt snout bream and grass carp (Supplementary Table 6). Additionally, scaffold_11,425 of a size of 16,606 bp, was found to be of mitochondrial origin, with 13 mRNAs, 22 tRNAs, and 2 rRNAs. Evolution of more complex eukaryotic organisms was impossible without gene duplication (Ohno, 1970), and analysis of duplicated genes in the rohu genome revealed 6,798 (26%) genes with more than one copy, comparable to the numbers observed for channel catfish (Liu et al., 2016) and zebrafish (Howe et al., 2013).

### Comparative Genome Analysis

**Figure 1:**
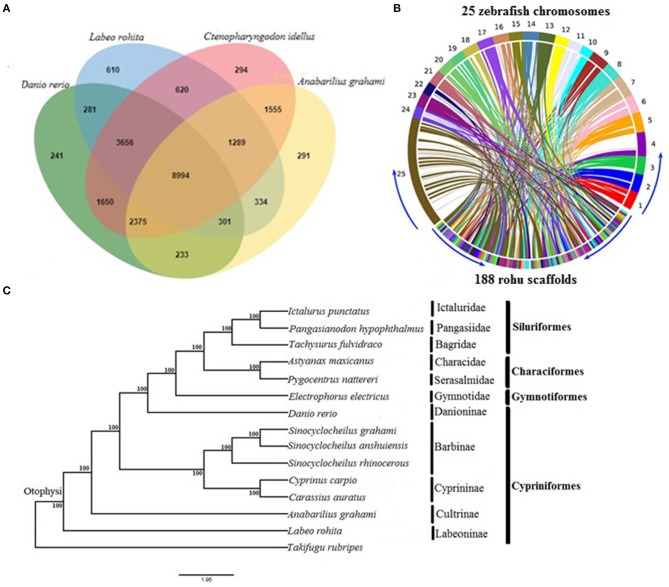
Comparative genomics of rohu **(A)** Venn diagram showing orthologous gene clusters among four diploid cyprinids, *Labeo rohita, Anabarilius grahami, Ctenopharyngodon idellus*, and *Danio rerio*. **(B)** Synteny conservation between rohu and zebrafish using Symap. The genome view is depicted by Circos plot where 25 zebrafish chromosomes (1 to 25) are shown in upper side and 188 largest scaffolds of rohu in the lower side of the ring. The connecting ribbons indicate the location of conserved synteny blocks between the two species. **(C)** Phylogenetic relationships of *Labeo rohita* with 13 other otophysans, inferred from 335 single-copy orthologous genes (protein sequences). Otophysan orders, families and subfamilies are identified by vertical bars against species names. ML bootstrap values are shown at the nodes.

The orthologous gene family analysis in diploid cyprinids, *C. idellus, A. grahami*, and *D. rerio* using, OrthoVenn resulted in a total of 22,724 clusters (rohu, 16,085; zebrafish, 17,731; white minnow, 15,372; grass carp, 20,433 orthologous clusters and 20,034 single-copy gene clusters) (Supplementary Table 8). A total of 8,994 orthologs are shared by all four species, with 1,669 species-specific gene clusters. Rohu and grass carp share the highest number of clusters (14,559), followed by rohu and zebrafish (13,232 clusters) and rohu and white minnow shared 10,918 (Figure 1A). Synteny between *L. rohita* and *D. rerio* was observed to be well-conserved (Figure 1B), as evidenced from synteny analysis between rohu scaffolds and zebrafish chromosomes.

### Whole-Genome Resequencing and SNP Discovery

Genome-wide SNP discovery using the NGS approach is straightforward and involves assembly of low depth sequencing data, followed by mapping of reads to a reference sequence, leading to variant calling. In contrast to livestock species, breeding programmes in the aquaculture sector have been slower to adopt genomics tools, mainly due to the paucity of genomic resources such as linkage maps, SNP arrays and reference genomes for important cultivable fish species. For species such as rainbow trout, salmon, and common carp, genomic selection (GS) and genome-wide association studies (GWAS) are being performed to improve the accuracy and speed of selective breeding for important performance traits (Bangera et al., 2017; Vallejo et al., 2018). To capture the variations in the rohu genome, low-depth resequencing of 10 wild rohu populations comprising thirty individuals was performed using Illumina Nextseq 500, which generated 60 Gb sequence data (40-fold coverage) of rohu genome. To improve the accuracy of SNP calling, three programs, SAMtools, VarScan, and FreeBayes, were used in the present study generating 4.95 million SNPs. The number of SNPs ranged from 380,991 to 679,963 in each population, and the number of common SNPs between any two populations ranged from 100,743 to 200,764. Identification of SNP markers has recently been carried out for several teleost species e.g., common carp, rainbow trout and greater amberjack (Xu J. et al., 2014; Palti et al., 2015; Araki et al., 2018). However, due to lack of SNP resources, SNP panels and arrays are not available for rohu carp. Thus, the SNPs identified from riverine populations of rohu in the present investigation, provide a valuable resource for undertaking genome wide association studies, genomic selection, population genomics and fine-mapping of QTLs in this species.

### Phylogenetic Relationship of Rohu Carp Within Otophysi

The phylogenetic position of *L. rohita* within Otophysi, revealed that rohu carp (Labeoninae) was at a position equidistant to the other species in the Otophysi clade, forming a sister group.

All the six families and four subfamilies under the four otophysan lineages were recovered as monophyletic groups (Figure 1C). Several hypotheses have been offered to discuss the evolutionary history of Otophysi. Characiformes was found to be a sister group to Gymnotiformes (Rosen et al., 1970); some authors argued for a sister group between Siluriformes and Gymnotiformes (Fink and Fink, 1981), whereas others found Characiformes to be paraphyletic (Nakatani et al., 2011). Our results reveal Characiformes, comprising the families Characidae and Serasalmidae, to be monophyletic, and together with Siluriformes, it forms a sister group with Gymnotiformes. This is in agreement with one of the tree topologies (Ha08) reported earlier (Nakatani et al., 2011). Classifications based on families and subfamilies are essential for diverse groups, such as Otophysi, when drawing taxonomic and evolutionary conclusions. Our results of sub familial relationships analysis within Cypriniformes are in agreement with recent studies (Xu P. et al., 2014; Jiang et al., 2018).

In summary, we report here the draft genome of rohu carp and associated genomics resources. Performing phylogenetic analysis, we show that rohu forms a sister group relationship with all remaining otophysans. The draft genome of rohu and SNPs generated in the present study represent essential resource for genetic improvement of important performance traits in this species. Besides, the information generated will provide foundation for future research in evolutionary biology and comparative genomics.

## Data Availability Statement

The datasets generated for this study can be found in the GenBank with the Bioproject id: PRJNA437789 and Acc no: QBIY00000000. RNAseq data have been submitted as SRA files or available online (SRR6987066, SRR6987067, SRR6987068, SRR7027731, SRR7027730, SRR7027732, GSE27994 and SRA051586). SNP information has been submitted to European Variation Archives with Accession number and link as follows: PRJEB36724 (https://www.ebi.ac.uk/ena/data/view/PRJEB36724).

## Ethics Statement

All the experiments in the present study were carried out in accordance with the guidelines for control and supervision of experiments on animals by the Government of India and approved by Institutional Animal Ethics Committee (AEC) of ICAR-CIFA.

## Author Contributions

PD, NN, BK, PJ, and JJ conceived the project and designed the objectives. LS, PM, SD, AB, SP, and KD did the sample selection, rearing, collection of tissues, and DNA/RNA isolation. High-throughput sequencing and data generation was performed by PD, LS, CJ, PK, and PM. *De novo* assembly and annotation were performed by CJ, BK, RK, MS, TS, AH, NP, AB, SD, SA, MP, SS, DK, MI, SJ, NK, and MR. Comparative and evolutionary analysis were carried out by PD, LS, AB, and SD. PD, LS, SD, and AB also performed MS writing.

## Conflict of Interest

The authors declare that the research was conducted in the absence of any commercial or financial relationships that could be construed as a potential conflict of interest.
