# Supplementary material for: De novo Assembly and Genome-Wide SNP Discovery in Rohu Carp, Labeo rohita
Source: Front Genet. 2020 Apr 21;11:386. doi: 10.3389/fgene.2020.00386 (PMC7186481; doi:10.3389/fgene.2020.00386)
Supplement: Supplementary file 1 [file Data_Sheet_1.doc]

**Platforms included**

**454 GS FLX**

**Illumina (MiSeq)**

**Illumina (NextSeq 500)**

**Ion Torrent PGM**

**(170.52Gb)**

**Platforms included**

**PacBio**

**(12.6Gb)**

***Labeo rohita* Genome Assembly Strategy**

**Assembly Assembly**

**(MaSuRCA Assembler) Illumina corrected PacBio data**

**(Canu Assembler)**

**259,627 Contigs**

**20,911 Contigs**

**Scaffolding in SSPACE Scaffolding in SSPACE**

**& gap closing**

**216,126 Scaffolds**

**17,972 scaffolds**

**Scaffold merging & Gap Closing**

**147,061 Scaffolds**

**Filtering of >2000bp scaffolds**

**Supplementary Fig. 1**: Schematic representation of assembly strategy of *Labeo rohita* genome.


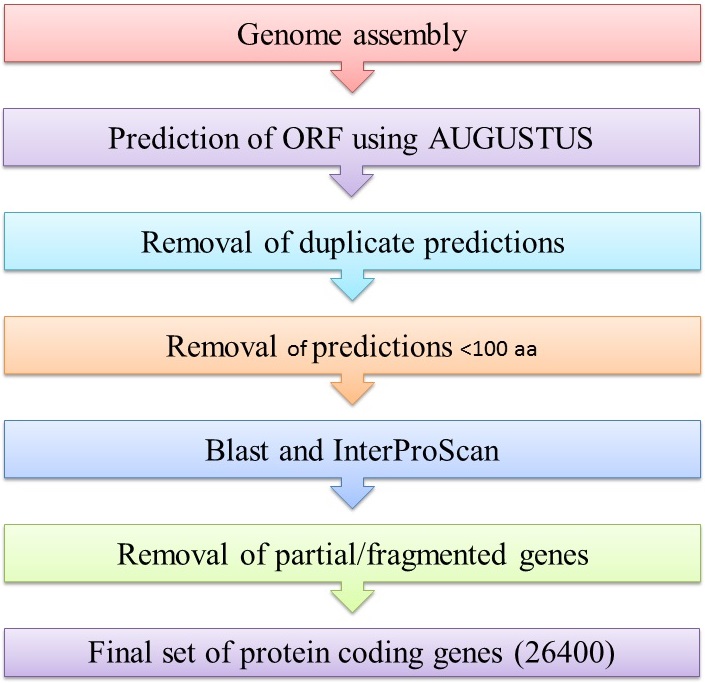


**Supplementary Fig. 2:** Prediction of protein coding genes in the genome of *Labeo rohita*

**Supplementary Fig. 3:** A 21-mer profile generated using Jellyfish for the complete assembly

**Supplementary Fig. 4:** 21-mer distribution profile for all the paired-end data of Illumina demonstrating a local maximum at around 32 kmer frequency. The plot shows the typical distribution of kmers in reads.

**Supplementary Fig. 5:** Illumina data mapped with assembly and plotting of coverage depth graphs. All different coverage plotted against number of bases including coverage=1 [there is a normal distribution with a long tail/skew towards right side].

**Supplementary Fig. 6:** All different coverage plotted against number of bases excluding coverage=1[there is a normal distribution with a long tail/skew towards right side].

**Supplementary Fig. 7:** All different coverage plotted against number of bases excluding coverage=1 and only coverage < 250 are plotted for better visibility of bell-shape [there is a normal distribution with a long tail/skew towards right side].

**Similar plots of 10 largest scaffolds demonstrating bell-shaped distribution (Fig. 8-17 below).**

**Supplementary Fig. 8: Scaffold_11923**

**Supplementary Fig. 9: Scaffold_11958**

**Supplementary Fig. 10: Scaffold_12186**

**Supplementary Fig.11: Scaffold_12347**

**Supplementary Fig. 12: Scaffold_12674**

**Supplementary Fig. 13: Scaffold_12728**

**Supplementary Fig. 14: Scaffold_12899**

**Supplementary Fig. 15: Scaffold_13169**

**Supplementary Fig. 16: Scaffold_13381**

**Supplementary Fig. 17: Scaffold_13465**

**Supplementary Table 1:** Summary of NGS platforms and size selected libraries used for sequencing, and data points generated in *Labeo rohita* genome

| **Illumina (MiSeq)** | | **Illumina (NextSeq 500)** | | **454 GS FLX** | | **Ion Torrent PGM** | | **PacBio-Sequel** | |
| --- | --- | --- | --- | --- | --- | --- | --- | --- | --- |
| **Library** | **Data (Gb)** | **Library** | **Data (Gb)** | **Library** | **Data (Gb)** | **Library** | **Data (Gb)** | **Library** | **Data (Gb)** |
| PE-200-400 bp | 3.12 | PE- 350-450 bp | 57.00 | SE- 450 bp | 2.31 | SE - 200 bp | 0.83 | 15-20 kb (Sequel-2 SMRT Cell) | 12.40 |
| PE-350-450 bp | 6.02 | PE -550-650 bp | 53.80 | MP-3 kb | 1.16 |  |  |  |  |
| PE-500-600 bp | 4.17 | MP_3 kb | 18.93 | MP-20 kb | 0.42 |  |  |  |  |
| PE-550-650 bp | 8.24 | MP_6 kb | 12.72 |  |  |  |  |  |  |
| MP-2-4 kb | 0.29 |  |  |  |  |  |  |  |  |
| MP-4-6 kb | 0.20 |  |  |  |  |  |  |  |  |
| MP-6-8 kb | 0.86 |  |  |  |  |  |  |  |  |
| MP-8-10 kb | 0.36 |  |  |  |  |  |  |  |  |
| MP-10-12 kb | 0.29 |  |  |  |  |  |  |  |  |
| **Total** | **23.55** |  | **142.45** |  | **3.89** |  | **0.83** |  | **12.40** |
| **Total Data = 183.12Gb** | | | | | | | | | |

SE- Single-End, PE- Paired-End, MP -Mate-Pair

**Supplementary Table 2:** Assembly quality of rohu genome compared with few published genomes

| **Serial Number** | **Species** | **Contigs N50** | **Scaffold N50** | **Genome size (Gb)** | **Coverage**  **Obtained (%)** |
| --- | --- | --- | --- | --- | --- |
| 1 | *Scophthalmus maximus* | 31.2kb | 4.3 Mb | 568 Mb | 95 |
| 2 | *Lates calcarifer* | 1.06 Mb | 1.19 Mb | 670 Mb | 99 |
| 3 | *Scleropages  formosus* | 30.7 kb | 5.9 Mb | 900 Mb | 94 |
| 4 | *Ctenopharyngodon idellus* | 40.78 kb | 6.45 Mb | 1.00 Gb | 96 |
| 5 | *Anabarilius graham* | 26.4 kb | 4.41Mb | 1.01Gb | 98 |
| 6 | *Labeo rohita* | 30.6 kb | 1.9 Mb | 1.50 Gb | 95 |

**Supplementary Table 3 :** Anchoring of *Labeo rohita* scaffolds into SNP map

| **LGs** | **Number of Loci** | **Number of  scaffolds anchored** | **Scaffold Size (MB)** |
| --- | --- | --- | --- |
| LG1 | 126 | 34 | 42.7 |
| LG2 | 100 | 18 | 30.4 |
| LG3 | 105 | 29 | 52.7 |
| LG4 | 140 | 37 | 76.8 |
| LG5 | 152 | 27 | 43.5 |
| LG6 | 120 | 20 | 31.7 |
| LG7 | 234 | 53 | 70.7 |
| LG8 | 123 | 30 | 58.7 |
| LG9 | 124 | 23 | 56.5 |
| LG10 | 130 | 23 | 52.5 |
| LG11 | 116 | 17 | 37.2 |
| LG12 | 100 | 19 | 42.7 |
| LG13 | 89 | 31 | 64.3 |
| LG14 | 101 | 23 | 31.6 |
| LG15 | 158 | 37 | 82.8 |
| LG16 | 96 | 26 | 43.8 |
| LG17 | 163 | 24 | 39.6 |
| LG18 | 139 | 27 | 45.9 |
| LG19 | 113 | 12 | 24.7 |
| LG20 | 148 | 43 | 51.8 |
| LG21 | 135 | 22 | 35.6 |
| LG22 | 152 | 30 | 54.2 |
| LG23 | 122 | 19 | 28.1 |
| LG24 | 103 | 23 | 43.9 |
| LG25 | 104 | 20 | 35.9 |
| **Total** | **3193** | **667** | **1178.3** |

**Supplementary Table 4:**  Anchoring of *Labeo rohita* scaffolds into SSR map

| **Linkage Groups** | **No. of Loci** | **No. of Scaffolds anchored** |
| --- | --- | --- |
| LG1 | 8 | 6 |
| LG2 | 6 | 5 |
| LG3 | 9 | 7 |
| LG4 | 14 | 11 |
| LG5 | 3 | 3 |
| LG6 | 3 | 2 |
| LG7 | 9 | 8 |
| LG8 | 6 | 5 |
| LG9 | 7 | 6 |
| LG10 | 2 | 2 |
| LG11 | 10 | 7 |
| LG12 | 6 | 5 |
| LG13 | 1 | 1 |
| LG14 | 6 | 6 |
| LG15 | 7 | 7 |
| LG16 | 2 | 2 |
| LG17 | 3 | 3 |
| LG18 | 2 | 1 |
| LG19 | 3 | 2 |
| LG20 | 12 | 10 |
| LG21 | 7 | 4 |
| LG22 | 10 | 9 |
| LG23 | 2 | 2 |
| LG24 | 4 | 4 |
| LG25 | 4 | 3 |
| **Total** | **146** | **121** |

**Supplementary Table 5:** Repeat content in *Labeo rohita* genome

| **Repeat Element** | **No. of elements** | **Length occupied (bp)** | **Percentage of sequence** |
| --- | --- | --- | --- |
| SINEs | 19,065 | 2,804,621 | 0.33 |
| LINEs | 32,341 | 6,958,808 | 0.82 |
| LTR elements | 42,582 | 14,340,072 | 1.69 |
| DNA elements | 830,909 | 157,469,575 | 18.6 |
| Unclassified | 691,139 | 107,269,537 | 12.67 |
| Small RNA | 7,250 | 1,368,038 | 0.16 |
| Satellites | 137,098 | 33,124,580 | 3.91 |
| Simple repeats | 412,879 | 19,648,413 | 2.32 |
| Low complexity | 579,59 | 3,564,659 | 0.42 |

**Supplementary Table 6:** Comparison of rohu genome sequence with another five cyprinids

| **SL No** | **Species** | **Number of protein coding genes** | **Repeat element content (%)** | **GC percentage (%)** |
| --- | --- | --- | --- | --- |
| 1 | *Labeo rohita* | 26,400 | 40.63 | 36 |
| 2 | *Cyprinus carpio* | 52,610 | 31.3 | 37.1 |
| 3 | *Synocyclocheilus graham* | 42,109 | 40.09 | 38.7 |
| 4 | *Ctenopharyngodon idellus* | 27,263 | 38.06 | 37.4 |
| 5 | *Danio rerio* | 26,206 | 52.2 | 36.7 |
| 6 | *Megalobrama amblycephala* | 23,696 | 34.18 | 37.3 |

**Supplementary Table 7:** Distribution of microsatellite repeat motifs in *Labeo rohita* genome

| **Total number of sequences examined** | **Total number of identified SSRs** | **di-repeats** | **tri-repeats** | **tetra-repeats** | **penta-repeats** | **hexa-repeats** |
| --- | --- | --- | --- | --- | --- | --- |
| 13,623 | 557,193 | 293,400 | 132,688 | 100,083 | 29,117 | 1,905 |

**Supplementary Table 8:** Summary of OrthoVenn analysis among three diploid cyprinids, *Labeo rohita, Anabarilius graham, Ctenopharyngodon idellus* and *Danio rerio*.

| **Species** | **Proteins** | **Clusters** | **Singletons** |
| --- | --- | --- | --- |
| *Labeo rohita* | 26,400 | 16,085 | 2,598 |
| *Anabarilius grahami* | 23,906 | 15,372 | 4,750 |
| *Danio rerio* | 24,544 | 17,731 | 7,900 |
| *Ctenopharyngodon*  *idellus* | 32,811 | 20,433 | 9,194 |
